# Underwater Sensor Networks for Communication, Navigation, and Localization

**DOI:** 10.3390/s25030593

**Published:** 2025-01-21

**Authors:** Inam Ullah

**Affiliations:** Department of Computer Engineering, Gachon University, Sujeong-gu, Seongnam 13120, Republic of Korea; inam@gachon.ac.kr

## 1. Introduction

Around 70% of the Earth is covered by water, with numerous submerged locations that have yet to be monitored and supervised. Furthermore, there are numerous types of undersea environments. Advances in underwater sensors and sensor networks make these places more accessible since sensors are becoming less expensive, have greater computational abilities, and use less battery power. Every day, the number of applications for which they can be used expands. Autonomous underwater vehicles (AUVs) can potentially remove the need for humans to perform dangerous underwater duties such as coral planting and underwater research. Despite decades of development, most underwater robots are still linked by cables and cannot achieve full autonomy. Unmanned aerial vehicles, or AUVs in the air, have been the subject of tremendous research advancements and have become a popular platform for diverse sensing requirements. More research is needed to improve AUVs’ localization, navigation, and communication performance. This Special Issue will collect articles on the most recent applications, developments, and problems in underwater sensor nodes and networks.

## 2. Scope of Special Issue

Authors were encouraged to submit original papers that had not been submitted to another conference or journal. The state of the art, standards, implementations, running experiments, applications, fresh research proposals, and industrial case studies were all considered.

Topics that could be considered included, but were not limited to:

- Underwater sensor networks’ wireless communication;
- Underwater sensor networks’ communication, localization, and distributed localization;
- Three-dimensional localization and recursive localization;
- Underwater object detection, target tracking, gas data, and multimedia communication using autonomous underwater vehicles;
- Identification of living creatures (plants and animals) in the sea;
- Tracing and tracking the paths of underwater submarines;
- Databases and big data for underwater systems control;
- Autonomous underwater vehicle localization;
- Underwater Internet of Things (UIoT);
- Underwater acoustic, visible light, radio frequency, and magnetic communications;
- Underwater acoustics, machine learning, deep learning, and signal processing;
- Underwater digital twins, virtual reality, augmented reality, and mixed reality;
- Underwater signal and image processing, marine environment and marine sciences, saliency detection, and underwater/underground mining;
- Machine learning/deep learning-based sensors’ signal processing for autonomous underwater vehicles.

The articles in this Special Issue discuss underwater sensor networks used for communication, navigation, and localization. For instance, in Contribution 1, the authors examine how BS motion affects handover decision errors, which arise when AUVs incorrectly initiate handovers to unintended BSs due to BS motion. By utilizing the AUV–BS distance as a handover-triggering parameter, their analysis reveals a significant increase in decision errors within the overlapping regions when both the current and target BSs are in motion, especially when moving in the same direction. In addition, these errors intensify with the magnitude of BS motion and are exacerbated by smaller BS network radii. Based on these simulation results, the authors present an analytical framework that measures the influence of BS motion on the AUV–BS distance and provides strategic insights for refining underwater handover protocols, thereby enhancing operational reliability and service continuity in B-UWANs.

In Contribution 2, the authors present Underwater Multi-channel Medium Access Control with Cognitive Acoustics (UMMAC-CA) as a suitable channel access protocol for distributed UCANs. UMMAC-CA operates per frame, like the Multi-channel Medium Access Control with Cognitive Radios (MMAC-CR) designed for distributed cognitive radio networks, but with notable differences. It employs a pre-determined data transmission matrix to allow all nodes to access the channel without contention, thus reducing the channel access overhead. In addition, to mitigate the communication failures caused by randomly occurring interferers, UMMAC-CA allocates at least 50% of its frame time to interferer sensing. This is possible because of the fixed data transmission scheduling, which allows other nodes to sense interference while a specific node is transmitting data. The simulation results demonstrate that UMMAC-CA outperforms MMAC-CR across various metrics, including sensing time rate, controlling time rate, and throughput. In addition, except for when the data transmission time coefficient equals 1, the message overhead performance of UMMAC-CA is also superior to that of MMAC-CR. These results underscore the suitability of UMMAC-CA for use in challenging underwater applications requiring multi-channel cognitive communication within a distributed network architecture.

Contribution 3 addresses the limitations discussed above by proposing an underwater source location privacy protection scheme based on game theory under the scenario of multiple cooperating attackers (SLP-MACGT). First, a virtual coordinate system transformation method is proposed to conceal the real position of nodes to a certain extent. Second, the relay node selection strategy increases the diversity of the transmission paths, passive attacks by adversaries are resisted, and the privacy of source nodes is protected. Additionally, a secure data transmission technique utilizing fountain codes is employed to resist active attacks by adversaries, ensuring data integrity and enhancing data transmission stability. Finally, Nash equilibrium is achieved after the multi-round evolutionary game theory of source nodes and after multiple attackers adopt their strategies. The simulation experiments and performance evaluation verify the effectiveness and reliability of SLP-MACGT regarding aspects of the packet forwarding success rate, security time, delay, and energy consumption: the packet delivery rate average increases by 30%, security time is extended by at least 85%, and delay is reduced by at least 90% compared with SSLP, PP-LSPP, and MRGSLP.

In Contribution 4, a trust-aware and fuzzy logic-based reliable layering routing protocol (TAFLRLR) is proposed. In TAFLRLR, to avoid the problem of the void area and improve the transmission reliability, the candidate nodes of the next-hop forwarding nodes are determined according to the layers of neighbor nodes. Moreover, a fuzzy logic-based trust evaluation mechanism (FLTEM) is provided, which employs the fuzzy comprehensive evaluation decision model to calculate the comprehensive trust value for underwater sensor nodes. Further, the node density of a candidate node and its comprehensive trust value is taken as the input of a fuzzy control system, the forwarding probability (FP) of the node is taken as the output, and the candidate node with the highest FP is selected as the best forwarding node. The simulation results illustrate the superiority and effectiveness of TAFLRLR in terms of energy efficiency, routing reliability, and transmission reliability.

Contribution 5 proposes a multichannel deep convolutional neural network (MDCNN) linked to a VGG to achieve multi-source (multi-domain) underwater image enhancement. The designed MDCNN feeds data from different domains into separate channels and implements parameters by linking VGGs, improving the model’s domain adaptation. In addition, to optimize performance, multi-domain image perception loss functions, multilabel soft edge loss for specific image enhancement tasks, pixel-level loss, and external monitoring loss for edge sharpness preprocessing are proposed. These loss functions are set to effectively enhance the structural and textural similarity of underwater images effectively. A series of qualitative and quantitative experiments demonstrate that our model is superior to the state-of-the-art Shallow UWnet in terms of UIQM, and the performance evaluation conducted on different datasets increased by 0.11 on average.

In Contribution 6, the authors discuss intrusion-detection systems (IDSs), which play a crucial role in identifying and preventing cyber hazards within IoT networks. However, developing an efficient and rapid IDS system for detecting cyber-attacks remains a challenging area of research. Moreover, IDS datasets contain multiple features, so feature selection (FS) is required to design an effective and timely IDS. The FS procedure seeks to eliminate irrelevant and redundant features from large IDS datasets, thereby improving the IDS’s overall performance. In this paper, the authors propose a hybrid wrapper-based feature-selection algorithm based on the concepts of the Cellular Automata (CA) engine and Tabu Search (TS)-based aspiration criteria. The authors used a Random Forest (RF) ensemble learning classifier to evaluate the fitness of the selected features. The proposed algorithm, CAT-S, was tested on the TON_IoT dataset. The simulation results demonstrate that the proposed algorithm, CAT-S, enhances the classification accuracy while reducing the number of features and the false positive rate.

Finally, Contribution 7 focuses on underwater wireless sensor networks (UWSNs) that have gained prominence in wireless sensor technology, discussing resource-limited sensor nodes deployed in challenging underwater environments. To address challenges like power consumption, network lifetime, node deployment, topology, and propagation delays, cooperative transmission protocols like co-operative (Co-UWSN) and co-operative energy-efficient routing (CEER) have been proposed. These protocols utilize broadcast capabilities and neighbor head node (NHN) selection for cooperative routing. This research introduces NBEER, a novel neighbor-based energy-efficient routing protocol tailored for UWSNs. NBEER aims to surpass the limitations of Co-UWSN and CEER by optimizing NHNS and cooperative mechanisms to achieve load-balancing and enhance network performance. They evaluated NBEER against Co-UWSN and CEER through comprehensive MATLAB simulations, demonstrating its superior performance across various metrics. NBEER significantly maximizes end-to-end delay, reduces energy consumption, improves the packet delivery ratio, extends the network lifetime, and enhances the total received packets analysis compared to the existing protocols.

## 3. Conclusions

As Guest Editor, I am proud to introduce this excellent Special Issue (SI). I believe that the articles featured in this Special Issue will constitute a significant resource for academics and professionals within the scientific community, providing insights and improvements that are both compelling and beneficial. I wish to express our sincere appreciation to the editorial staff of the *Sensors* journal for their steadfast support during the editorial process. Their commitment to overseeing the preliminary arrangements and submission processing, as well as their thorough evaluation of the manuscripts, was crucial in guaranteeing the superior quality of this Special Issue. I also appreciate the authors’ outstanding contributions, demonstrating their pioneering research and inventive solutions. Furthermore, I extend our heartfelt gratitude to the reviewers, whose prompt and insightful input significantly improved the quality of the published works. It has been a privilege to collaborate with such a skilled and dedicated team, and I aspire for this SI to motivate future study and advancements in the discipline.

